# The association of symptoms, pulmonary function test and computed tomography in interstitial lung disease at the onset of connective tissue disease: an observational study with artificial intelligence analysis of high-resolution computed tomography

**DOI:** 10.1007/s00296-025-05934-z

**Published:** 2025-08-12

**Authors:** Tobias Hoffmann, Ulf Teichgräber, Luis Benedict Brüheim, Bianca Lassen-Schmidt, Diane Renz, Tobias Weise, Martin Krämer, Peter Oelzner, Joachim Böttcher, Felix Güttler, Gunter Wolf, Alexander Pfeil

**Affiliations:** 1 Department of Internal Medicine III, Center of Rheumatology, Jena University Hospital, Friedrich Schiller University Jena, Am Klinikum 1, 07747 Jena, Germany; 2 Institute of Diagnostic and Interventional Radiology, Jena University Hospital, Friedrich Schiller University Jena, Jena, Germany; 3 Fraunhofer Institute for Digital Medicine MEVIS, Bremen, Germany; 4 Institute of Diagnostic and Interventional Radiology, Department of Pediatric Radiology, Hannover Medical School, Hannover, Germany; 5 BioControl Jena GmbH, Jena, Germany

**Keywords:** Inflammatory rheumatic diseases, Interstitial lung disease, Pulmonary symptoms, Artificial intelligence, HRCT, Quantification, Rheumatic diseases, Connective tissue diseases, Lung diseases, Interstitial, Tomography, X-ray computed, AI-based quantification of pulmonary HRCT

## Abstract

Interstitial lung disease (ILD) is a common and serious organ manifestation in patients with connective tissue disease (CTD), but it is uncertain whether there is a difference in ILD between symptomatic and asymptomatic patients. Therefore, we conducted a study to evaluate differences in the extent of ILD based on radiological findings between symptomatic/asymptomatic patients, using an artificial intelligence (AI)-based quantification of pulmonary high-resolution computed tomography (AIpqHRCT). Within the study, 67 cross-sectional HRCT datasets and clinical data (including pulmonary function test) of consecutively patients (mean age: 57.1 ± 14.7 years, woman *n* = 45; 67.2%) with both, initial diagnosis of CTD, with systemic sclerosis being the most frequent (*n* = 21, 31.3%), and ILD (all without immunosuppressive therapy), were analysed using AIqpHRCT. 25.4% (*n* = 17) of the patients with ILD at initial diagnosis of CTD had no pulmonary symptoms. Regarding the baseline characteristics (age, gender, disease), there were no significant difference between the symptomatic and asymptomatic group. The pulmonary function test (PFT) revealed the following mean values (%predicted) in the symptomatic and asymptomatic group, respectively: Forced vital capacity (FVC) 69.4 ± 17.4% versus 86.1 ± 15.8% (*p* = 0.001), and diffusing capacity of the lung for carbon monoxide (DLCO) 49.7 ± 17.9% versus 60.0 ± 15.8% (*p* = 0.043). AIqpHRCT data showed a significant higher amount of high attenuated volume (HAV) (14.8 ± 11.0% versus 8.9 ± 3.9%; *p* = 0.021) and reticulations (5.4 ± 8.7% versus 1.4 ± 1.5%; *p* = 0.035) in symptomatic patients. A quarter of patients with ILD at the time of initial CTD diagnosis had no pulmonary symptoms, showing DLCO were reduced in both groups. Also, AIqpHRCT demonstrated clinically relevant ILD in asymptomatic patients. These results underline the importance of an early risk adapted screening for ILD also in asymptomatic CTD patients, as ILD is associated with increased mortality.

## Introduction

Lung involvement is the most common and serious organ manifestation in patients with connective tissue disease (CTD) [1, 2]. CTD summarizes numerous inflammatory rheumatic diseases, including systemic sclerosis (SSc), Sjögren´s disease, mixed connective tissue disease (MCTD), idiopathic inflammatory myopathy (IIM) or rheumatoid arthritis (RA). The type of pulmonary complications can vary, but the most typical manifestation is interstitial lung disease (CTD-ILD) and granuloma in small vessel vasculitis or sarcoidosis [3]. The likelihood of pulmonary involvement differs between the CTD-entities with the highest prevalence in patients with systemic sclerosis (SSc; 44–50%), mixed connective tissue disease (MCTD; 25.0%), and IIM (33–50%) [2, 4].

The clinical manifestations and findings show a wide array of symptoms ranging from (exertional) dyspnoea, cough, sputum or sclerosiphonia to digital clubbing [1]. However, up to 32% of patients are asymptomatic early in the disease course [5].

ILD is the leading cause of SSc-related mortality: 35% of the SSc-related deaths were attributed to ILD [6]. Given the significant increased mortality, early diagnosis and severity assessment of CTD-ILD is essential, especially in the light of innovative therapeutic strategies [7–10].

In most cases, the diagnosis of CTD-ILD is made based on the combination of clinical symptoms, physical examination, non-invasive diagnostic tools (e.g. pulmonary function test [PFT]), and high-resolution computed tomography (HRCT) which is the main diagnostic tool to assess ILD [1, 2, 5, 8, 11, 12]. The morphological findings of HRCT are characterized according to the definition of the Fleischner Society and the ATS/ERS [12–14]. Different HRCT patterns are differentiated, the most common being non-specific interstitial pneumonie (NSIP) or usual interstitial pneumonie (UIP) in CTD [4], but also different morphological structures/ ILD features can be characterized on HRCT, like ground glass opacities (GGO), consolidations or reticulations.

However, several studies have shown substantial inter-observer variations even among experienced thoracic radiologists in visual assessment [12, 14, 15]. In addition, there is no precise definition of the extent of fibrotic changes for CTD-ILD to be present [1]. Furthermore, a validated and established scoring system for the verification of CTD-ILD does not exist, since visual scores are inconsistent and relatively irreproducible, and even experienced chest radiologists can eventually struggle with differential diagnosis [16]. In order to address this, computer-based analysis was introduced years ago, including automated techniques such as histogram- and pattern/texture-based analyses, as well as deep learning–based methods [16]. The traditional methods already demonstrated its potential compared to qualitatively assessed in disease severity assessment and treatment response in CTD-ILD [17, 18]. Artificial intelligence (AI) tools, such as AIqpHRCT, a new technique uses the browser-based platform SATORI (Segmentation and Annotation TOol for Radiomics and Deep LearnIng), have already shown high performance in segmentation [19–21] and can automatically and reliable quantify and visualize the extent based on each lung zone and generate and visualize the non-fibrotic and fibrotic ILD features individually [15, 21]. AI-based scoring systems also show moderately to strong correlations with visual scoring systems [22], but they can also predict mortality risk in idiopathic pulmonary fibrosis [23]. In CTD, AI based analysis are limited, but most examined in SSc. Previous studies demonstrated an increase of SSc-ILD lesions as the disease progresses [24], but also the correlation of AI-measured ILD extent with important functional parameter (FVC or TLC) in CTD and SSc [21, 25].

The objective of this cross-sectional study was (1) to evaluate the prevalence of pulmonary symptoms in patients with the initial diagnosis of ILD in CTD, since until now, it is uncertain whether there is a difference in ILD between asymptomatic/symptomatic patients, and (2) to analyse differences in the extent of ILD between symptomatic and asymptomatic patients using a pulmonary assessment including PFT, HRCT, and AIpqHRCT. To the best of our knowledge, no study has yet been published that analysed the difference of pulmonary symptomatic and asymptomatic CTD patients, on an AI approach.

## Methods

### Study design and participants

This study analysed 67 cross-sectional HRCT datasets of 22 men (32.8%) and 45 women (67.2%) with ILD at initial diagnosis of CTD between 2010 and 2022 (systemic sclerosis *n* = 21, 31.3%; IIM *n* = 23, 34.3%; Sjögren´s disease *n* = 9, 13.4%; SLE *n* = 7, 10.4%; MCTD *n* = 5, 7.5% and rheumatoid arthritis [RA] *n* = 2, 3.0%) (Table 1). The data was collected retrospectively. The exclusion criteria were defined as already known diagnosed CTD as well as immunosuppressive (incl. glucocorticoids) or antifibrotic treatment. In consequence, the diagnosis of ILD, diagnosed through risk adapted screening [5, 26], was simultaneous with the initial diagnosis of CTD in all patients.

**Table 1:** Baseline characteristics of symptomatic and asymptomatic patients with CTD-ILD

Baseline characteristics			ILD		*p*-value
			Symptomatic	Asymptomatic	
Number of patients			*n* = 50 (74.6%)	*n* = 17 (25.4%)	
Age			58.2 ± 11.4 years	54.0 ± 16.4 years	*p* = 0.502
Gender					*p* = 0.961
Female			*n* = 33 (66.0%)	*n* = 12 (70.6%)	
Male			*n* = 17 (34.0%)	*n* = 5 (29.4%)	
Diseases (CTD)					
SSc			*n* = 15 (30.0%)	*n* = 6 (35.3%)	*p* = 0.917
Sjögren’s syndrome			*n* = 7 (14.0%)	*n* = 2(11.8%)	*p* = 1.000
MCTD			*n* = 2 (4.0%)	*n* = 3 (16.6%)	*p* = 0.188
SLE			*n* = 5 (10.0%)	*n* = 2 (11.8%)	*p* = 1.000
IIM			*n* = 20 (40.0%)	*n* = 3 (17.6%)	*p* = 0.167
Anti Jo1-syndrome			*n* = 11 (22.0%)	*n* = 1 (5.9%)	*p* = 0.258
Dermatomyositis			*n* = 7 (14.0%)	*n* = 1 (5.9%)	*p* = 0.646
Polymyositis			*n* = 2 (4.0%)	*n* = 1 (5.9%)	*p* = 1.000
RA			*n* = 1 (2.0%)	*n* = 1 (5.9%)	*p* = 1.000
Pulmonary symptoms					
Dyspnoea			*n* = 43 (86.0%)	*n* = 0 (0.0%)	n/a
Sklerosiphonia			*n* = 31 (62.0%)	*n* = 0 (0.0%)	n/a
Cough			*n* = 24 (48.0%)	*n* = 0 (0.0%)	n/a
Sputum			*n* = 14 (28.0%)	*n* = 0 (0.0%)	n/a
Pulmonary comorbidities					
Emphysema			*n* = 1 (2.0%)	*n* = 0 (0.0%)	*p* = 1.000
COPD			*n* = 2 (4.0%)	*n* = 0 (0.0%)	*p* = 0.990
Smoking status					
Active			*n* = 6 (12.0%)	*n* = 5 (29.4%)	*p* = 0.236
Ex-smoker			*n* = 13 (26.0%)	*n* = 3 (17.6%)	
Pack years			11.1 ± 15.0	9.17 ± 12.4	*p* = 0.860

*CTD* connective tissue disease; *ILD* interstitial lung disease; *MCTD* mixed connective tissue disease; *RA* rheumatoid arthritis; *SLE* systemic lupus erythematosus; *SSc* systemic sclerosis; *IIM* idiopathic inflammatory myopathy

**Table 2:** Result of pulmonary function tests in symptomatic and asymptomatic patients with CTD-ILD

PFT (%predicted)	ILD		*p*-value
	Symptomatic	Asymptomatic	
Forced expiratory volume per second (FEV_1_)	73.0 ± 17.2	86.9 ± 13.4	*p* = 0.002
Forced vital capacity (FVC)	69.4 ± 17.4	86.1 ± 15.8	*p* = 0.001
Total lung capacity (TLC)	74.5 ± 16.4	84.8 ± 13.9	*p* = 0.024
Diffusing capacity of the lung for carbon monoxide (DLCO)	49.7 ± 17.9	60.0 ± 15.8	*p* = 0.043
Transfer coefficient (DLCO/VA)	71.7 ± 23.8	75.8 ± 18.1	*p* = 0.325

*ILD* interstitial lung disease; *CTD* inflammatory rheumatic disease

**Table 3:** ILD patterns in HRCT in symptomatic and asymptomatic patients with CTD-ILD

ILD patterns	ILD		*p*-value
	Symptomatic	Asymptomatic	
Pure ground glass opacities (GGO)	*n* = 20 (40.0%)	*n* = 10 (58.8%)	*p* = 0.286
Non-specific interstitial pneumonia (NSIP)	*n* = 27 (54.0%)	*n* = 4 (23.5%)	*p* = 0.058
Usual interstitial pneumonia (UIP)	*n* = 3 (6.0%)	*n* = 1 (5.9%)	*p* = 1.000
Other ILD pattern	*n* = 0 (0.0%)	*n* = 2 (11.8%)	*p* = 0.102

*ILD* interstitial lung disease; *CTD* inflammatory rheumatic disease

**Table 4:** AIqpHRCT data in symptomatic and asymptomatic patients with CTD-ILD

AIqpHRCT data		ILD		*p*-value
		Symptomatic	Asymptomatic	
Volumetry in litre		3.93 ± 1.28	4.24 ± 1.39	*p* = 0.407
Emphysema in %		4.96 ± 4.47	7.06 ± 6.65	*p* = 0.485
High attenuated volume (HAV) in %		14.80 ± 11.00	8.91 ± 3.91	*p* = 0.021
Overall extent in %		16.30 ± 17.80	8.72 ± 10.00	*p* = 0.053
Ground glass opacities (GGO) in %		10.50 ± 10.30	7.10 ± 8.73	*p* = 0.096
Reticulations in %		5.41 ± 8.65	1.44 ± 1.52	*p* = 0.035
Honeycombing in %		0.40 ± 1.35	0.18 ± 0.53	*p* = 0.684

*ILD* interstitial lung disease; *CTD* inflammatory rheumatic disease

All participants had been divided into two groups regarding the pulmonary symptoms dyspnoea, cough, sputum, and sclerosiphonia on physical examination. Group A included patients with none of these symptoms, and participants in group B had at least one of these symptoms.

In addition, pulmonary comorbidities (COPD and severe emphysema) and smoking status were documented.

### Procedures

All patients were diagnosed with newly CTD based on a comprehensive rheumatologic assessment. In addition, participants underwent a standardised pulmonary assessment including the quantification of pulmonary symptoms, PFT, and HRCT of the lungs. The diagnosis of ILD was performed in a consensus panel by rheumatologists, pulmonologists, and radiologists using clinical, laboratory, and imaging findings; no patient has been previously evaluated for ILD. All participants were examined at the Department of Internal Medicine III, Rheumatology and Osteology, University Hospital Jena/Germany.

#### PFT

PFT encompassed forced expiratory volume in 1 s (FEV_1_), forced vital capacity (FVC), total lung capacity (TLC), diffusing capacity of the lung for carbon monoxide (DLCO), and transfer coefficient (DLCO/VA).

#### HRCT

Multi-slice computed tomography was used for all HRCT images (General Electric Healthcare Technologies, Revolution, Waukesha, Wisconsin; USA) with a primary slice thickness of 0.625 mm (*n* = 38) and 1 mm (*n* = 29) and a reconstructed slice thickness of 2.5 mm–3.0/4.0 mm. All scans were analysed with respect to parenchymal changes (especially ground glass opacities [GGO], non-specific interstitial pneumonia [NSIP, usual interstitial pneumonia [UIP] or other ILD pattern) by two chest radiologists and a rheumatologist according to the recommendations/criteria by the American Thoracic Society (ATS)/European Respiratory Society (ESC) and the Fleischner Society White Paper [12–14].

#### AI-based quantification of pulmonary HRCT (AIpqHRCT)

The AIpqHRCT-based tool SATORI is a browser-based platform for curating medical data, developed by the Fraunhofer Institute for Digital Medicine Mevis, Bremen/Germany [27].

**Fig. 1:**
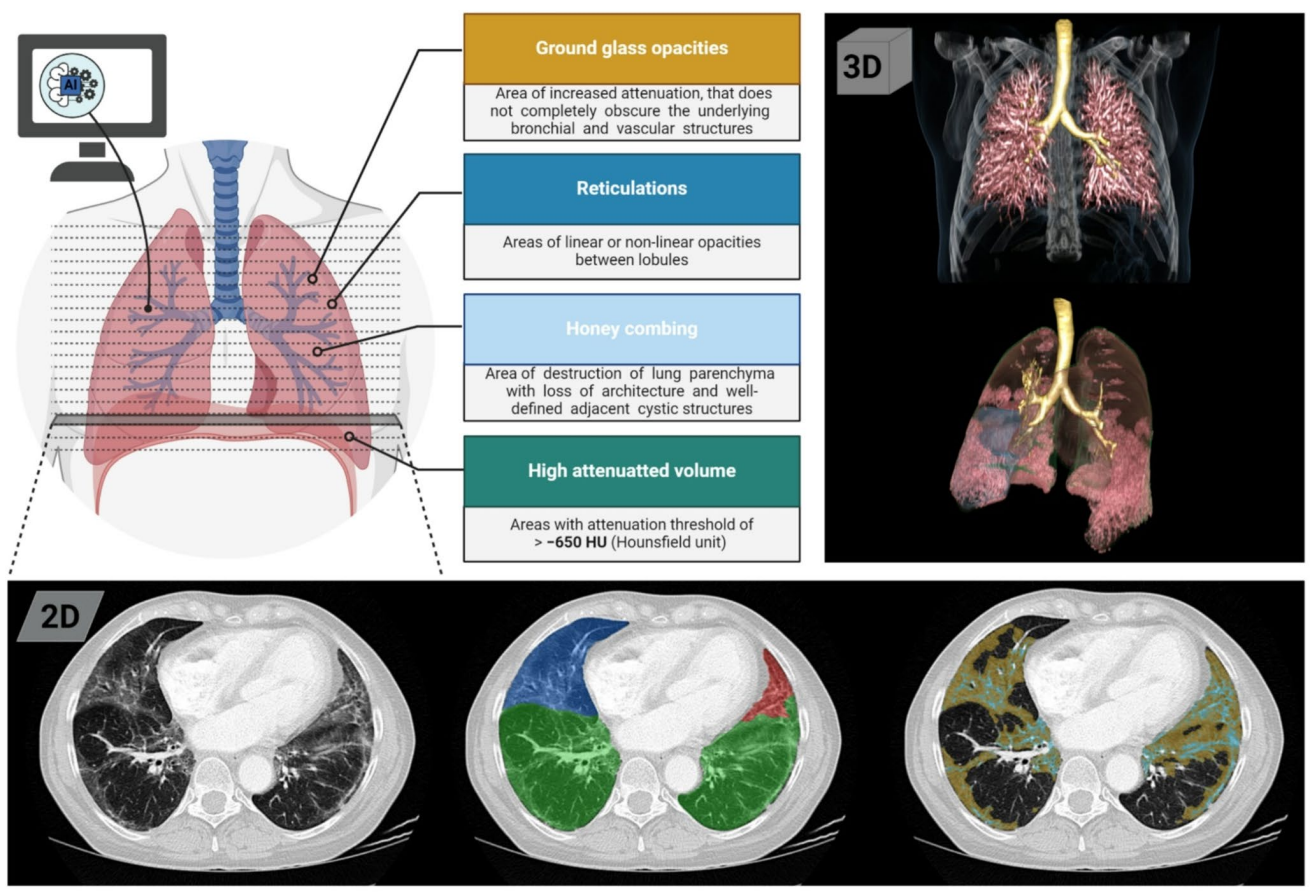
Mode of operation of AIqpHRCT for the quantification of ILD in CTD, with 2-dimensional and 3-dimensional screen views of the quantified lung pathologies. Created in BioRender. Agreement number: GO28KUF4F3; Pfeil, A. (2025) https://BioRender.com/xs0krd2. *ILD* interstitial lung disease; *CTD* inflammatory rheumatic disease

In accordance with international recommendations, we used AIpqHRCT for an automated quantification of GGO [5, 6, 28], NSIP with the radiographic features of GGO and reticulations [5, 8, 11], and UIP with the radiographic features of GGO, reticulations, and honeycombing [5, 8, 11] (Fig. 1).

Since SATORI does not allow automated quantification of honeycombing and traction bronchiectasis, a manual segmentation was carried out to capture honeycombing. All SATORI examinations were performed using a 2.5 mm–3.0/4.0 mm slice thickness.

**Fig. 2:**
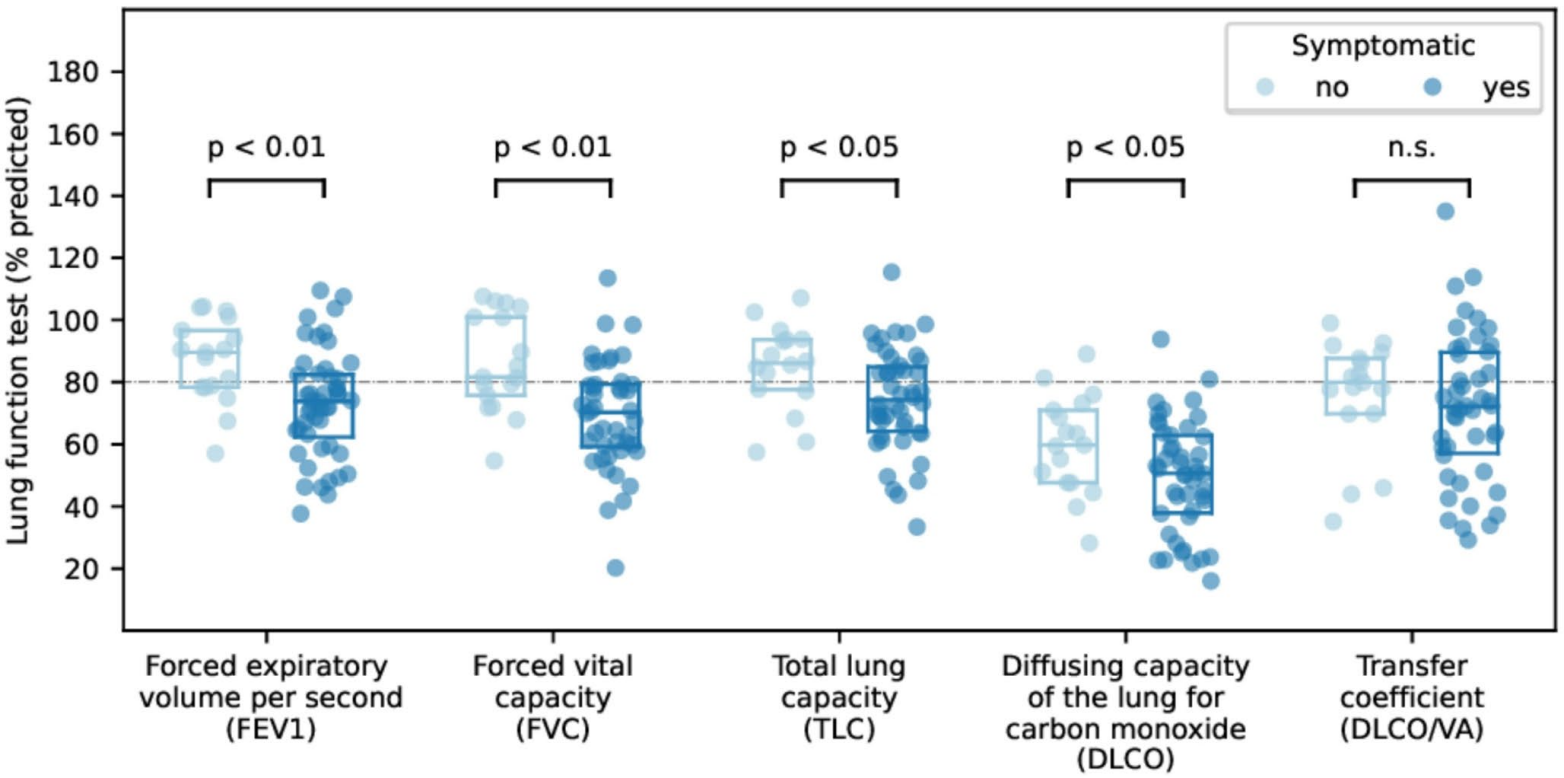
PFT in symptomatic and asymptomatic CTD-patients with initial diagnosis of ILD; 80% predicted as reference line (·−·). *ILD* interstitial lung disease; *CTD* inflammatory rheumatic disease

**Fig. 3:**
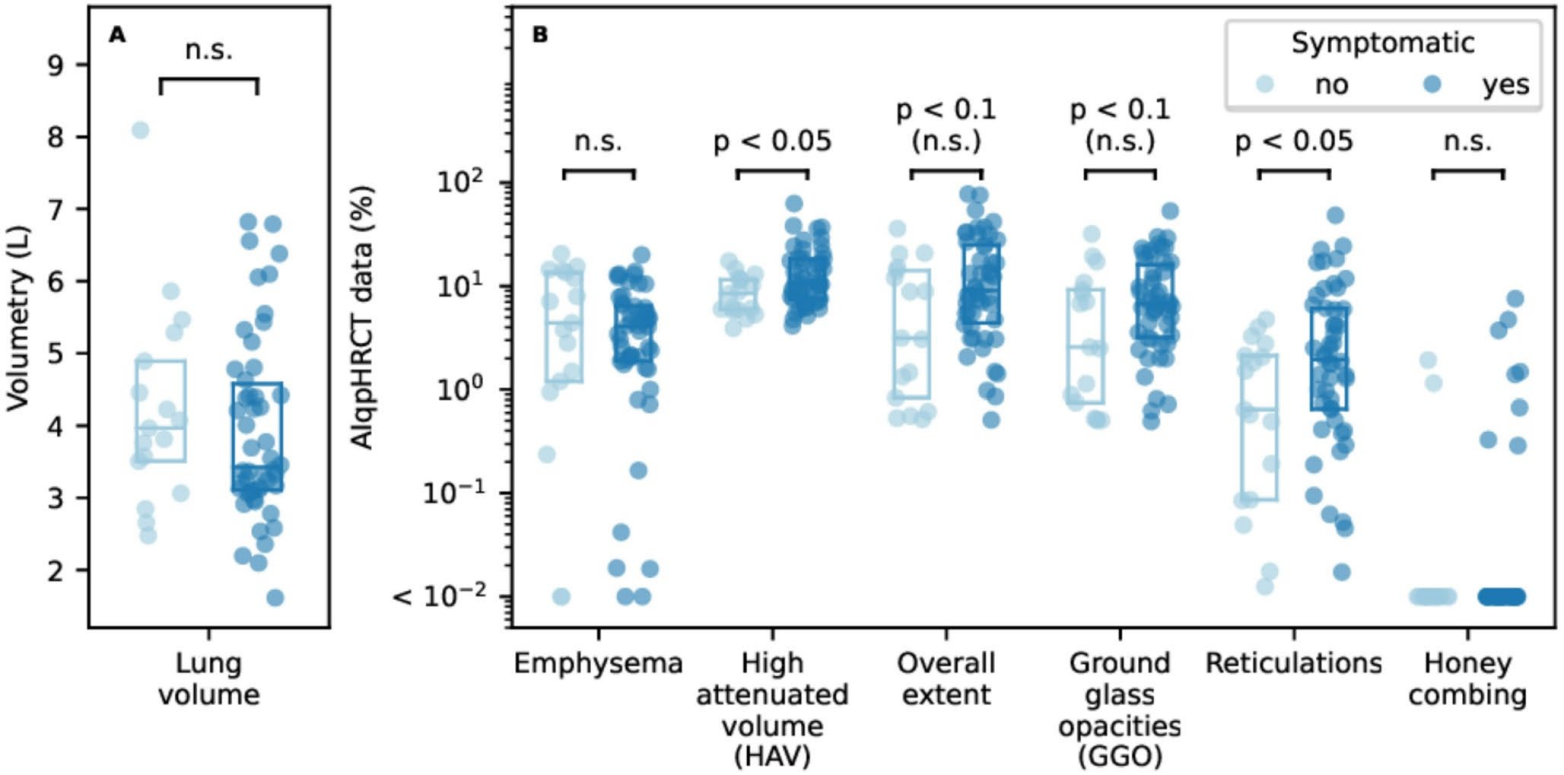
AIqpHRCT data in in symptomatic and asymptomatic patients with initial diagnosis of ILD in CTD patients; **A**– volumetry in litre (L) and **B**– ILD abnormalities in percent (%).* ILD* interstitial lung disease;* CTD* inflammatory rheumatic disease

**Fig. 4:**
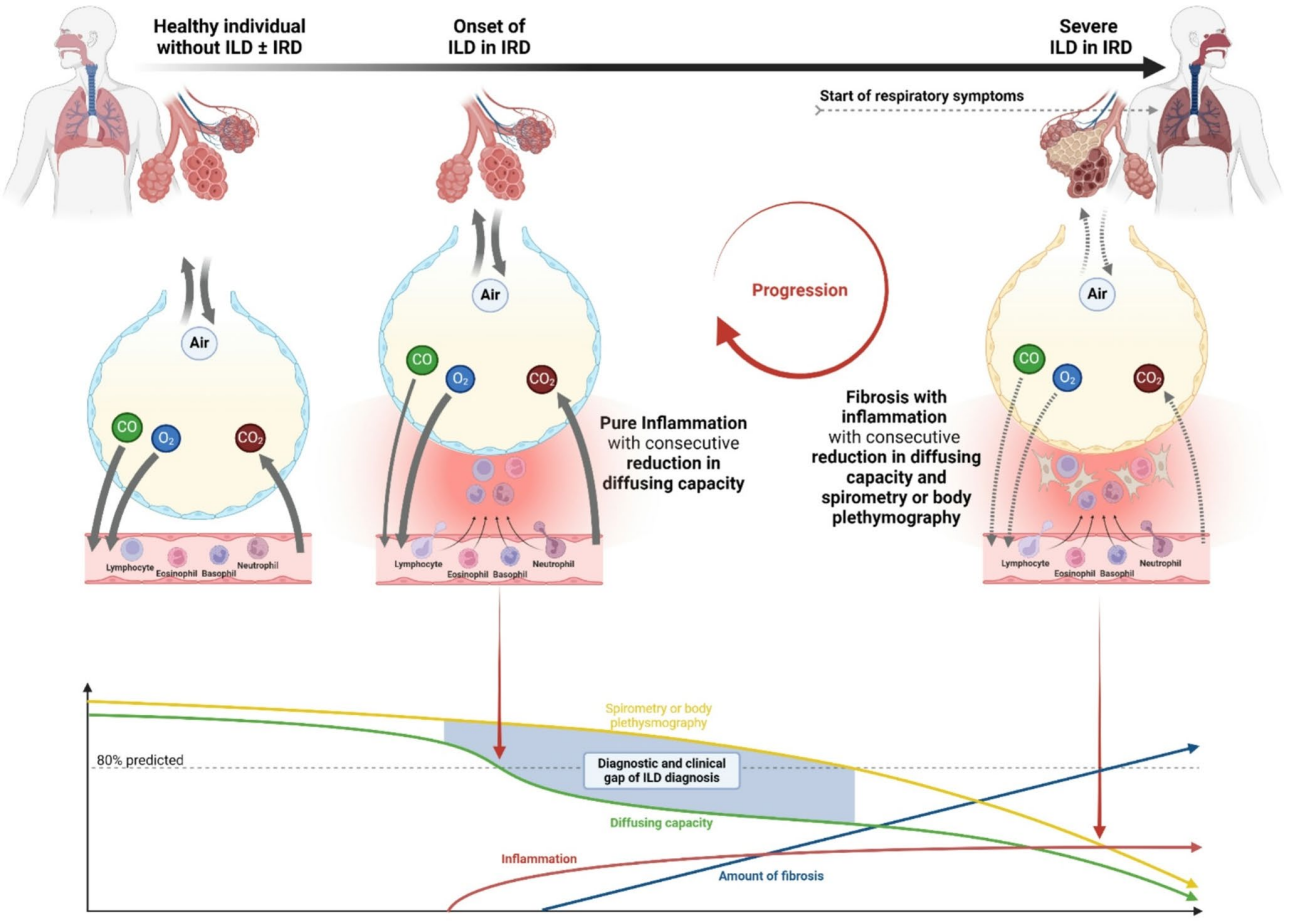
Pathophysiological, diagnostic, and therapeutic aspects of ILD in CTD patients. Created in BioRender. Agreement number: IJ28KUFYFL; Pfeil, A. (2025) https://BioRender.com/prozmq2. *ILD* interstitial lung disease; *CTD* inflammatory rheumatic disease

#### AIqpHRCT workflow

The HRCT images were obtained from the hospital picture archiving and communication system (PACS) and pseudonymized using an in-house developed digital imaging and communications in medicine (DICOM) pseudonymization platform before the transfer into the RACOON infrastructure. Afterwards, the corresponding HRCT images were accessed via the web-based SATORI interface and lung parenchymal changes were quantified [29]. AIqpHRCT quantified automated lung volume (volumetry), GGO, reticulations, high attenuation lung volume (HAV; a histogram-based measurement of lung fibrosis via hounsfield units), and emphysema. In addition, a manual segmentation of honeycombing was carried out in each of the slices. These quantitative data were then extracted from SATORI on an analysis/case basis and converted into an Excel file for further statistical analysis.

### Statistical analysis

Data collection and documentation was carried out using Microsoft Excel^®^ (Microsoft Windows, Redmond Washington, USA). We performed the descriptive data analysis and data processing by using the programming language Python (version 3.10.13) and the additional packages numpy (version 1.26.0), pandas (version 2.1.1), and scipy (version 1.11.4). Data visualisation was carried out using the packages matplotlib (version 3.8.0) and seaborn (version 0.13.0).

The respective statistical significance level of group differences between symptomatic and asymptomatic patients (see Tables 1, 2, 3 and 4) was calculated using the Mann-Whitney U test (scipy.stats.mannwhitneyu) for continuous variables as well as the Chi-Square test (scipy.stats.chi2_contingency) for categorical variables. P-values < 0.05 were considered statistically significant.

## Results

### Pulmonary symptoms

74.6% of patients showed at least one pulmonary symptom and 58.2% two or more symptoms (Table 2). The most relevant symptoms and findings were dyspnoea (86.0%), sclerosiphonia (62.0%), cough (48.0%), and sputum (28.0%) (Table 1). 69.8% (*n* = 30) of patients showed mild to moderate dyspnoea and 30.2% (*n* = 13) severe to very severe dyspnoea, measured according to the ATS score [30]. There was no significant difference in age, gender, or disease distribution, as well as in pulmonary comorbidities, or smoking status and pack years between group A (no ILD-symptoms) and group B (≥ 1 symptom).

### PFT

PFT revealed the following mean values (%predicted) in symptomatic and asymptomatic group (Table 2; Fig. 2): FVC 69.4 ± 17.4% versus 86.1 ± 15.8%, total lung capacity (TLC) 74.5 ± 16.4% versus 84.8 ± 13.9%, FEV1 73.0 ± 17.2 versus 86.9 ± 13.4, DLCO 49.7 ± 17.9% versus 60.0 ± 15.8%, and DLCO/VA 71.7 ± 23.8% versus 75.8 ± 18.1%. The analysis showed significant differences between group A and B in FEV_1_ (*p* = 0.002), FVC (*p* = 0.001), TLC (*p* = 0.024), and DLCO (*p* = 0.043), but no significant difference in the transfer coefficient (DLCO/VA; *p* = 0.325).

### Qualitative analysis of pulmonary HRCT

The qualitative and expert-based analysis of the pulmonary HRCT revealed the following ILD pattern in symptomatic and asymptomatic patients (Table 3): Pure GGO *n* = 20 (40.0%) versus *n* = 10 (58.8%), NSIP *n* = 27 (54.0%) versus *n* = 4 (23.5%), and UIP *n* = 3 (6.0%) versus *n* = 1 (5.9%). There were no significant differences in the distribution of the analysed ILD patterns between group A and B (GGO *p* = 0.286; NSIP *p* = 0.058; UIP *p* = 1.000), but a trend towards a reduced number of NSIP in asymptomatic patients (*p* = 0.058).

### Quantitative analysis with AIqpHRCT

AIqpHRCT data revealed a significant higher amount of HAV (14.8 ± 11.0% vs. 8.9 ± 3.9%; *p* = 0.021) and reticulations (5.4 ± 8.7% versus 1.4 ± 1.5%; *p* = 0.035) in symptomatic patients, but no significance difference in volumetry (3.93 ± 1.28 L vs. 4.24 ± 1.39 L; *p* = 0.407), emphysema (4.96 ± 4.47% vs. 7.06 ± 6.65%; *p* = 0.485), and GGO (10.5 ± 10.3% vs. 7.1 ± 8.7%; *p* = 0.096) (Table 4; Fig. 3). The same was seen for honey combing (in 0.40 ± 1.35% vs. 0.18 ± 0.53; *p* = 0.684).

## Discussion

The aim of our study was to evaluate the frequency of pulmonary symptoms in patients with the initial diagnosis of ILD in CTD and to find potential differences in the extent of ILD between symptomatic and asymptomatic patients using a pulmonary assessment including PFT, HRCT, and AIpqHRCT.

### Pulmonary symptoms

In our study, 25.4% of newly diagnosed CTD-patients had no pulmonary symptoms at the initial diagnosis of ILD, which is in accordance with the data of a previously performed case-control study [5]. We found no significant differences in baseline characteristics such as age, gender, comorbidities, or smoking, or in disease distribution and ILD patterns. Misinterpretation based on these parameters can therefore be largely excluded.

Comparison with the literature is hampered by the lack of other studies of pulmonary symptoms at initial diagnosis of ILD in CTD. However, an analysis of 448 patients with SSc and an average disease duration of seven years showed that of the 80% patients with ILD, only 60.5% had respiratory symptoms [28]. In another retrospective study by Marie et al., 30% of IIM patients with ILD were asymptomatic [31]. Other publications demonstrated 14–71% of CTD-ILD patients with pulmonary symptoms at the initial clinical manifestation or diagnosis [32, 33]. This confirms that a substantial proportion of CTD-patients with ILD has no respiratory symptoms at the time of initial diagnosis or during the disease. In addition, Hu et al. showed in SSc patients with and without ILD that baseline presence of respiratory symptoms was associated with worse prognosis [28].

### PFT and qualitative analysis of pulmonary HRCT

Pathological changes of PFT parameters are known and extensively evaluated as surrogate parameters for mortality in patients with idiopathic pulmonary fibrosis (IPF), but also in CTD-patients with ILD [34–37]. PFT is a simple and cost-effective method of screening, monitoring, and prognostic assessment of ILD in CTD [1, 26]. Our current study demonstrated significant lower mean values differences in FEV_1_, FVC, TLC, and DLCO in symptomatic CTD-ILD patients. However, mean values of DLCO were also reduced (< 80% predicted) in patients without pulmonary symptoms. A review of the literature reveals an absence of publications comparing pulmonary symptomatic and asymptomatic CTD-ILD patients, but an interpretation on pathophysiological principles remains possible.

Any disease leading to a thickening of the pulmonary membrane, but also morbidities like pulmonary hypertension, results in a decreased diffusion coefficient and a reduced DLCO [38]. Histologically, reduced DLCO in ILD patients can be explained by inflammation and/or fibrosis and consequent thickening of the alveolar-capillary space [39] (Fig. 4). In systemic sclerosis genetic predisposition, risk factors and environmental insult lead to ILD and progression over time, through chronic endothelial and epithelial injury, inflammation, fibroblast activity and the increase of extracellular matrix [40–42]. In disease progression both, alveolo-capillary space thickening and the impairment of diffusion capacity exacerbates, which was seen in comparison of the symptomatic and asymptomatic CTD-ILD patients. In addition, the increasing fibrotic tissue causes lung retraction with volume loss and ventilatory compromise, as reflected by a decrease in FVC, TLC, and FEV_1_ [39]. Our findings underline DLCO as the key marker in PFT parameter for the early detection of CTD-ILD as previously shown by our group in the context of comparing patients with and without ILD in CTD [5]. Regarding the distribution of the ILD-patterns GGO, NSIP, and UIP between CTD-ILD patients with and without pulmonary symptoms have seen no significant differences.

### Quantitative analysis with AIqpHRCT

AIqpHRCT data revealed a significant higher amount of HAV and reticulations as markers of fibrotic ILD features in symptomatic patients, but no statistically significant difference in GGO. However, asymptomatic patients had a relevant mean ILD involvement of 8.9% of the lungs (HAV) in comparison to 14.8% in symptomatic patients. Comparative analyses are not available and the histological correlation of inflammation and fibrotic tissue with GGO, reticulations and honey combing in HRCT data is limited [40, 41]. But Verleden et al. demonstrated that regions with reticulation on HRCT generally had greater fibrosis at histopathologic analysis in early ILD [43]. Moreover, honeycombing correlate histologically best with bronchiolectasis and cysts, as a sign of fibrotic change [44]. Based on these morphological-histological correlations, the study demonstrated that CTD-ILD patients with pulmonary symptoms have a more severe ILD, with higher proportion of fibrotic lung tissue.

In a previous study AIqpHRCT demonstrated high reliability for detecting ILD features on HRCT in CTD, but also significant correlations between the extent of ILD and important lung function parameters (FVC, DLCO and TLC) [21]. Previous AI-based HRCT studies analysed disease progression [25] or characterise CTD-ILD [24], but also the correlation of AI-measured ILD extent with important functional parameter (FVC or TLC) in CTD and SSc [21, 25]. This study provides a novel comparison between pulmonary symptomatic and asymptomatic CTD-ILD patients and their functional and morphological changes, but also the tight pathophysiological connection between PFT and HRCT. We revealed a clinically relevant ILD burden of 8.9% in asymptomatic CTD-ILD patients, which would be an indication for immunosuppressive therapy, especially in the context of inflammatory cell profile in immunological bronchioalveolar lavage or biopsy [45].

These aspects implying direct consequences for clinical practice, especially for screening of CTD-ILD. CTD-ILD represents a continuum from mild to severe. The data suggest that pulmonary symptoms develop alongside a higher degree of fibrosis as seen on HRCT, indicating a “clinical gap” in ILD diagnosis (Fig. 4). Furthermore, these considerations result in a “diagnostic gap” between DLCO and conventional PFT parameters (Fig. 4). Therefore, it is unclear whether symptomatic patients have had ILD/CTD for a longer period of time, or if they are showing signs of rapid progression and may therefore require more intensive treatment. This finding could be indicative of the presence of two distinct patient groups, or the identification of patients at varying stages of ILD progression. The outcome of these patients should be evaluated in further studies.

Potential limitations of our study are the monocentric, retrospective design and the limited number of patients. However, due to the retrospective and consecutively design, the data are particularly reflective of daily clinical practice.

## Conclusion

A quarter of patients with an initial diagnosis of both CTD and ILD are asymptomatic despite radiologically relevant ILD manifestations. These pulmonary asymptomatic patients show significantly higher functional parameters in terms of FEV1, FVC, and TLC in PFT compared to pulmonary symptomatic CTD-ILD patients. However, among these functional parameters, only DLCO showed a decrease < 80% in pulmonary asymptomatic patients, resulting in a “diagnostic gap” at initial diagnosis of ILD between the PFT parameters for ventilation (FVC, TLC, FEV1) and diffusion (DLCO). Moreover, AIqpHRCT data revealed a significant lower amount of ILD extent, measured by HAV, in asymptomatic (8.9%) to symptomatic (14.8%) patients, nevertheless being an indication for immunosuppressive therapy. The data suggest that pulmonary symptoms develop alongside a higher degree of fibrosis as seen on HRCT, indicating a “clinical gap” in ILD diagnosis. At present, it remains to be seen whether there is a difference in outcome between these two patient groups and whether different treatment regimens are indicated, further research is required. Our findings emphasise the importance of improved and earlier screening for ILD also in asymptomatic CTD patients.

The indication for ILD screening depends on the specific characteristics of the patient and their individual risk factors, if there are any uncertainties, indication for screening should proceed [26]. The findings of this study also indicate the necessity of performing a PFT, which should include a DLCO measurement, while decrease < 80% may indicate ILD even if there are no pulmonary symptoms. Furthermore, it is imperative to systematically record pulmonary symptoms, as their presence may be indicative of more pronounced ILD. However, the absence of symptoms does not rule out ILD; careful diagnostics must always be carried out.

## Data Availability

The data underlying this article will be shared on reasonable request to the corresponding author.

## Abbreviations

AI: Artificial intelligence
AIqpHRCT: AI-based quantification of pulmonary HRCT
ATS: American thoracic society
COPD: Chronic obstructive lung disease
CTD: Connective tissue disease
DICOM: Digital imaging and communications in medicine
DLCO: Diffusing capacity of the lung for carbon monoxide
DLCO/VA: Transfer coefficient
ERS: European respiratory society
FEV_1_: Forced expiratory volume per second
FVC: Forced vital capacity
GGO: Ground-glass opacity
HAV: High-attenuated volume
HRCT: High-resolution computed tomography
ILD: Interstitial lung disease
IPF: Idiopathic pulmonary fibrosis
NSIP: Non-specific interstitial pneumonia
PACS: Picture archiving and communication system
PFT: Pulmonary function test
RACOON: Radiological cooperative network
SATORI: Segmentation and annotation tool for radiomics and deep learning
TLC: Total lung capacity
UIP: Usual interstitial pneumonia
